# Quantification of dose accumulation using deformable image registration: Application in re‐irradiation of liver metastases using robotic radiosurgery

**DOI:** 10.1002/acm2.70278

**Published:** 2025-10-07

**Authors:** Ahamed Badusha Mohamed Yoosuf, Mohd Zahri Abdul Aziz, Mohd Syahir Mansoor, Gokula Kumar Appalanaido, Salem Alshehri, Mamdouh Alqathami

**Affiliations:** ^1^ Department of Oncology Ministry of National Guard Health Affairs Riyadh Saudi Arabia; ^2^ Clinical Research King Abdullah International Medical Research Center (KAIMRC) Riyadh Saudi Arabia; ^3^ Advanced Management of Liver Malignancies Program Advanced Medical and Dental Institute, Universiti Sains Malaysia Penang Malaysia; ^4^ Radiotherapy Unit, Pusat Perubatan Universiti Sains Malaysia Advanced Medical and Dental Institute, Universiti Sains Malaysia Penang Malaysia; ^5^ Nuclear Medicine Unit, Pusat Perubatan Universiti Sains Malaysia, Advanced Medical and Dental Institute Universiti Sains Malaysia Penang Malaysia; ^6^ Radiological Sciences King Saud bin Abdulaziz University for Health Sciences Riyadh Saudi Arabia

**Keywords:** deformable image registration (DIR), dose accumulation, liver metastases, multiple re‐irradiation, robotic stereotactic radiosurgery

## Abstract

**Purpose:**

This study evaluates cumulative dose estimations using deformable image registration (DIR) in robotic stereotactic ablative radiotherapy (SABR)‐based multiple re‐irradiations for liver metastases. It highlights DIR's role and accuracy in adaptive radiotherapy to enhance treatment precision and reduce toxicity.

**Materials & Methods:**

A retrospective analysis was conducted on 22 patients (age: 42–80, median: 61 years) with liver metastases re‐irradiated using CyberKnife SABR (54 treatments) between June 2016 and February 2024. A comparative analysis of organs‐at‐risk (OAR) volumes was performed using contours derived from DIR and physical summation method to evaluate consistency between the two approaches. Dosimetric analysis involved accumulating doses to organs at risk using physical dose summation and DIR‐based dose summation. To standardize dose assessments, all radiation doses were converted into equivalent doses in 2 Gy fractions (EQD2) and biologically effective doses (BED). The DIR algorithm was quantitatively assessed using similarity indices, including Dice similarity coefficient (DSC) and Jaccard (JD) index.

**Results:**

The findings demonstrated that organs susceptible to motion, such as the liver and large bowel, exhibited greater variability in volume measurements when evaluated using physical summation. A significant reduction in maximum dose for the liver (*p* = 0.00) and chest wall (*p* = 0.05) was observed under DIR‐based dose accumulation (liver‐83.2 ± 28.0 Gy; chest wall‐66.9 ± 18.6 Gy) compared to physical summation (liver‐123.8 ± 55.6 Gy; chest wall‐82.9 ± 22.4 Gy), suggesting overestimation using physical summation. Among the analyzed structures, DIR showed high spatial accuracy for the heart, liver, and external body with Dice scores >  0.90 and Jaccard indices >  0.84, while lower agreement was noted for deformable organs such as the bowel with Dice scores <  0.43 and Jaccard indices <  0.29.

**Conclusions:**

DIR improved anatomical alignment and provides more anatomically consistent cumulative dose estimation in SABR re‐irradiation, particularly for OARs that are prone to deform. Integrating DIR into adaptive radiotherapy workflows can improve the estimation of dose to OARs while minimizing toxicity risks.

## 1 INTRODUCTION

Liver metastases frequently occur in solid organ cancers. Colorectal cancer, which drains to the liver through the portal circulation, is the common primary source of metastatic liver disease.^1^ The liver is also a frequent site for metastatic disease from pancreatic and breast cancers. The development of liver metastases was once thought to be an incurable disease state, but during the past 20 years, improvements in imaging, systemic treatment, surgery, and locally ablative techniques have shown that a more aggressive approach is necessary, particularly for patients with oligometastatic disease.^1^

While surgical resection is a preferred treatment, it is often limited by high recurrence rates and surgical complexities.^2^, ^3^ Stereotactic ablative body radiotherapy (SABR) has emerged as an effective noninvasive alternative, offering high precision and minimal toxicity.^4^, ^5^, ^6^ The CyberKnife system (Accuray Incorporated, Sunnyvale, CA, USA), a robotic image‐guided device, administers SBRT, monitors tumors while breathing, and adapts treatment in response to patient movements.^7^, ^8^ The CyberKnife robotic radiosurgery system, an advanced image‐guided technology, has demonstrated promising results in both primary and metastatic liver cancers by delivering high‐dose radiation with sub‐millimeter accuracy.^9^, ^10^ The effectiveness of re‐irradiation, particularly focusing on gastrointestinal (GI) and liver metastases, has been reported comprehensively in a recent review.^11^

Radiotherapy has advanced significantly, shifting from conventional target coverage to precision‐based treatment strategies that enhance tumor control while reducing damage to surrounding healthy tissues.^12^, ^13^, ^14^ Nearly half of all cancer patients receive radiotherapy at some stage, either as a curative or palliative approach.^15^, ^16^ The integration of advanced imaging, adaptive planning, and optimized radiation delivery has further improved survival and quality of life outcomes.^14^, ^17^

Re‐irradiation is increasingly utilized for managing recurrent or residual malignancies when other treatment options are limited.^18^ This therapeutic approach entails administering radiation to previously irradiated areas, offering a potential avenue for disease control and symptom palliation in cases where conventional treatment options may be limited.^19^ However, delivering additional radiation to previously treated areas poses significant challenges. Tissues exposed to prior irradiation exhibit reduced tolerance, making cumulative dose management crucial to minimize toxicity in organs at risk (OARs) such as the liver, GI structures, and adjacent organs. Multiple re‐irradiations, defined as three or more radiation treatments to the same anatomical site, further amplify these risks.^20^ The risk of complications and side effects increases with each successive re‐irradiation.^21^, ^22^ Tumor progression, post‐surgical anatomical changes, and respiratory motion introduce complexities in accurately summing radiation doses across multiple treatment sessions.

The traditional approach, referred to as physical dose summation, involves the direct addition of the dose received by individual OARs from each treatment without making any adjustments for anatomical changes or patient movement between treatments. This method is simple and requires minimal computational resources; however, this may lead to potential inaccuracies in dose estimations to OARs.^23^ Image registration algorithms play a crucial role in adaptive radiotherapy by aligning multiple images acquired at various times to a common coordinate system and quantifying changes within images.^24^ These algorithms are essential for planning radiotherapy, verifying treatment, automating OAR segmentation, and managing dose accumulation in the management of re‐irradiation.^25^, ^26^

Deformable image registration (DIR) provides a more precise approach by accommodating non‐linear deformations such as organ motion, swelling, and structural variations. DIR adapts prior dose distributions to real‐time anatomical changes, refining cumulative dose calculations and better estimating dose to OARs. Studies have demonstrated that DIR enhances accumulated dose estimation, leading to improved treatment outcomes and reduced normal tissue toxicity.^27^, ^28^, ^29^ The importance of accurately calculating and monitoring accumulated dose during re‐irradiation has been highlighted to optimize treatment planning and delivery.^30^, ^31^

This study aims to compare cumulative dose estimations for OARs using DIR as compared to conventional physical dose summation in CyberKnife‐based SABR for liver metastases, with a focus on quantifying its accuracy. By assessing the advantages of DIR in multiple re‐irradiation scenarios, this study addresses a critical gap in adaptive radiation therapy for recurrent liver cancer.

## 2 MATERIALS AND METHODS

### 2.1 Patient characteristics–liver metastases

The study retrospectively analyzed 22 patients treated with at least two SABR‐based re‐irradiation sessions between June 2016 and February 2024. Eligible patients had an Eastern Cooperative Oncology Group (ECOG) performance status of ≤2 and presented with 1–5 liver metastases. Patients were excluded if they had an ECOG score > 2, a tumor size ≥6 cm, more than five re‐irradiation treatments, or uncontrolled extrahepatic disease. Each re‐irradiation treatment was validated through disease restaging using whole‐body 18F‐fluorodeoxyglucose positron emission tomography/computed tomography (18FDG‐PET/CT) or liver MRI.

### 2.2 Robotic stereotactic radiosurgery system and treatment planning

All patients underwent SABR using the CyberKnife (Accuray, Sunnyvale, CA), a robotic radiosurgery platform that employs real‐time image guidance for precise tumor targeting. The system integrates a linear accelerator mounted on a robotic arm, enabling sub‐millimeter accuracy in radiation delivery.^32^ Treatment plans were generated using the Precision Treatment Planning System (TPS), which incorporates multimodal imaging, including computed tomography (CT), magnetic resonance imaging (MRI), and PET‐CT, to delineate both target volumes and OARs. The gross tumor volume (GTV) was defined using the most recent imaging before each re‐irradiation session, and a uniform 3‐mm planning target volume (PTV) margin was added to compensate for variations in setup and respiratory motion. Beginning with the second SBRT session, a DIR technique was employed to delineate the previously irradiated region in each treatment plan, which was then designated as an avoidance contour to reduce potential dose overlap.

### 2.3 Dose prescription and delivery using motion management

Fractionation schedules varied based on tumor characteristics and prior radiation exposure, with prescribed doses ranging from 18 to 60 Gy in 1–5 fractions. The treatment was delivered on an outpatient basis, with fractions scheduled every other day. To ensure precise tumor localization, three or more radio‐opaque fiducials were implanted in each patient under ultrasound guidance at least 7 days before CT simulation. Fiducials were placed following Accuray guidelines, and a multi‐slice CT scan (1.25‐mm slice thickness, end of exhale) was acquired. Real‐time respiratory motion tracking was also performed using the Synchrony respiratory tracking system, which continuously monitored fiducial movement and synchronized beam delivery accordingly. The cumulative dose constraints for each OAR were decided on a case‐by‐case basis based on published studies on SBRT methods and cumulative EQD2. Senior radiation oncologists who were board‐certified examined and approved each plan.

### 2.4 Dose accumulation workflow using DIR

**FIGURE 1:**
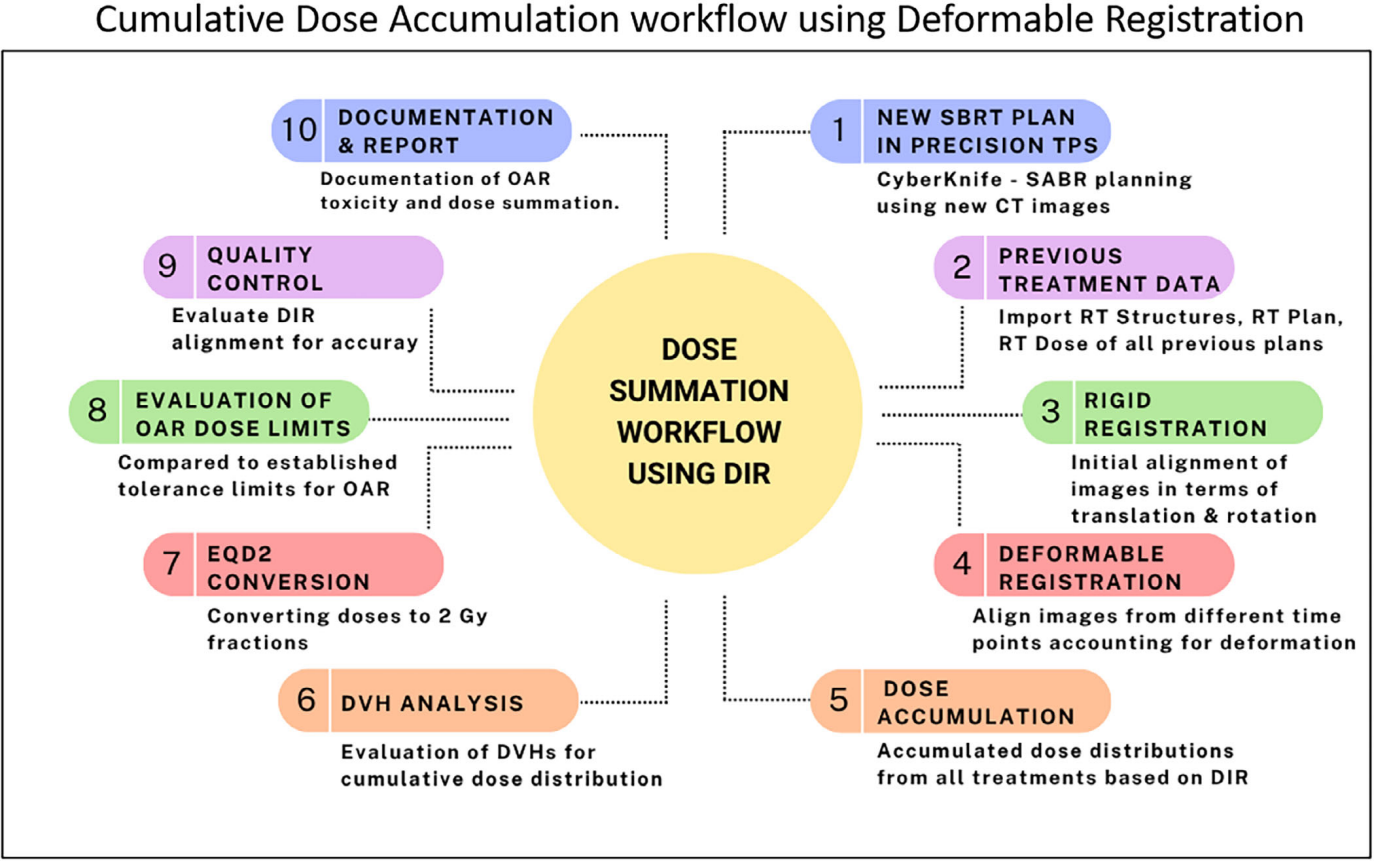
Dose summation workflow for re‐irradiation treatment planning.

DIR was implemented using MIM (version 6.6.8 MIM Software, Cleveland, OH, USA), an intensity‐based, free‐form registration algorithm designed to align images based on voxel‐based transformations. DIR accounts for non‐linear anatomical deformations, including organ shifts, tissue swelling, and respiratory‐induced motion. The Precision® TPS (version 4.0.2) was used to extract treatment plans, dose distributions, and imaging datasets, which were subsequently imported into MIM software for dose accumulation analysis. Initially, rigid registration was performed using bony landmarks to align sequential imaging datasets, followed by DIR‐based image registration to account for anatomical variations across multiple treatment sessions, and subsequently cumulative dose is calculated as shown in Figure 1. In contrast, the conventional physical dose summation method involved direct arithmetic summation of radiation doses from each treatment session without accounting for anatomical changes.^33^ The volume of each organ at risk and dose distribution was evaluated between physical and DIR‐based dose accumulation. Dose‐volume histograms (DVHs) were then generated from the summed plans for patients who underwent two or more courses of SABR, using the cumulative dose distributions.^34^

### 2.5 Biological dose evaluation and DVH analysis

To standardize dose assessments, all radiation doses achieved using DIR were converted into equivalent dose in 2 Gy fractions (EQD2) and biologically effective dose (BED) within the MIM software. EQD2 sum plans were computed utilizing the MIM software solutions for every re‐irradiation course. Following the addition of the number of fractions, the structures were compared using the α/β‐values (target–10 and OARs–3). The cumulative dose received by OARs was analyzed using established AAPM TG‐101 guidelines.^35^

### 2.6 Qualitative and quantitative assessment of DIR accuracy

DIR accuracy was evaluated through both qualitative and quantitative assessments. Visual validation techniques included overlay displays, checkerboard comparisons, and contour‐based alignment tools in MIM software.^36^ This qualitative assessment helps validate the registration results by correlating the contour with the anatomy on the secondary image.^37^, ^38^

Quantitative registration accuracy was evaluated using multiple metrics to ensure precise alignment of anatomical structures across imaging datasets from treatment plans at different timelines. To quantitatively determine the uncertainty of contour matching, the study employed Dice Similarity Coefficient (DSC) and Jaccard (JD) index between structures. The DSC was used to measure volumetric overlap between organ‐at‐risk (OAR) contours, with an optimal range of 0.8–0.9 as per AAPM TG‐132 guidelines.^26^ Similarly, the JD was used to evaluate local volume changes in deformed anatomical structures, where values near 1 indicate minimal distortion, and negative values may signal registration inaccuracies.

### 2.7 Statistics

Descriptive statistics were used to summarize patient demographics, registration metrics, and dosimetric parameters. To assess differences between DIR‐based dose summation and conventional physical dose summation, paired *t*‐tests were conducted, with statistical significance set at *p* < 0.05.

## 3 RESULTS

### 3.1 Patient and treatment characteristics

Table 1 illustrates the patient demographics and treatment characteristics for a total of 22 patients with liver metastases who underwent multiple rounds (*n* = 54 treatment sessions) of SABR using the CyberKnife system between June 2016 and February 2024. Patients who had received multiple courses of SABR presented with metachronous metastases in different liver lobes, rather than undergoing repeated treatment of the same lesion. Patients were categorized based on the location of their lesions prior to SBRT: those with lesions situated within the same liver segment (e.g., an initial lesion in segment 2 followed by a non‐local recurrence also in segment 2) and those with lesions in different, remote segments. Of the repeated SBRT cases, 2 involved re‐irradiation of the same segment, while 20 involved treatment of a separate segment. The patient cohort included individuals aged 42–80 years, with a median age of 61 years. The majority of patients (54.5%) were male, while 45.5% were female. The primary tumor diagnosis varied, with rectal cancer being the most common (*n* = 14, 64%), followed by kidney (*n* = 3, 13.6%) and other malignancies. All patients (*n* = 22) underwent at least two SABR sessions, while 33.3% (*n* = 8) received three treatments, and one patient had up to five courses of radiation therapy. The time interval between re‐irradiation sessions varied, with a median interval of 6 months (range: 0–35 months) between the first and second courses and a median interval of 5 months (range: 0–18 months) for subsequent treatments.

**TABLE 1:** Patient demographics and treatment details.

Patient characteristics (June 2016–Feb 2024)	*n*	%
**Gender**		
Male	12	51.7
Female	10	48.3
**Re‐irradiation treatment sessions**	52	
**Total number of lesions**	65 (Min 2; Max–5)	
**Age (Median & range)**	61 years (42–80 years)	
**Primary tumor**		
Rectum	14	38.3
Kidney	3	16.7
Sarcoma	2	11.7
Others	3	10.0
**Staging**		
Stage IV	37	61.6
Stage III	16	26.7
Stage II	4	6.7
Stage I	3	5.0
**Number of repeated radiotherapy (Re‐irradiation)**		
2	22	55.0
3	8	33.3
4	2	8.3
5	1	3.3
**Time interval between treatment (months)**		
1^st^–2^nd^ course	6 months (0–35)	
2^nd^–3^rd^ course	5 months (0–18)	
3^rd^–4^th^ course	6 months (0–10)	
**Prescription (Dose / fraction)**		
6000 cGy / 5#	5	9.6
5400 cGy / 3#	2	3.8
5000 cGy / 5#	18	34.6
4800 cGy / 3#	14	27.0
4000 cGy / 5#	4	7.7
3500 cGy / 5#	6	11.5
3000 cGy / 5#	3	5.8

Among the 52 treatment courses analyzed, the most frequently prescribed dose prescription was 5000 cGy delivered in 5 fractions (34.6%), followed by 4800 cGy in 3 fractions (27.0%). The highest dose prescription of 6000 cGy in 5 fractions was administered in 9.6% of cases, while the lowest dose of 3000 cGy in 5 fractions was prescribed for 5.8% of cases. Additionally, other dose prescriptions included 5400 cGy in 3 fractions (3.8%), 4000 cGy in 5 fractions (7.7%), and 3500 cGy in 5 fractions (11.5%).

### 3.2 OAR volume comparisons: DIR versus physical summation

**FIGURE 2:**
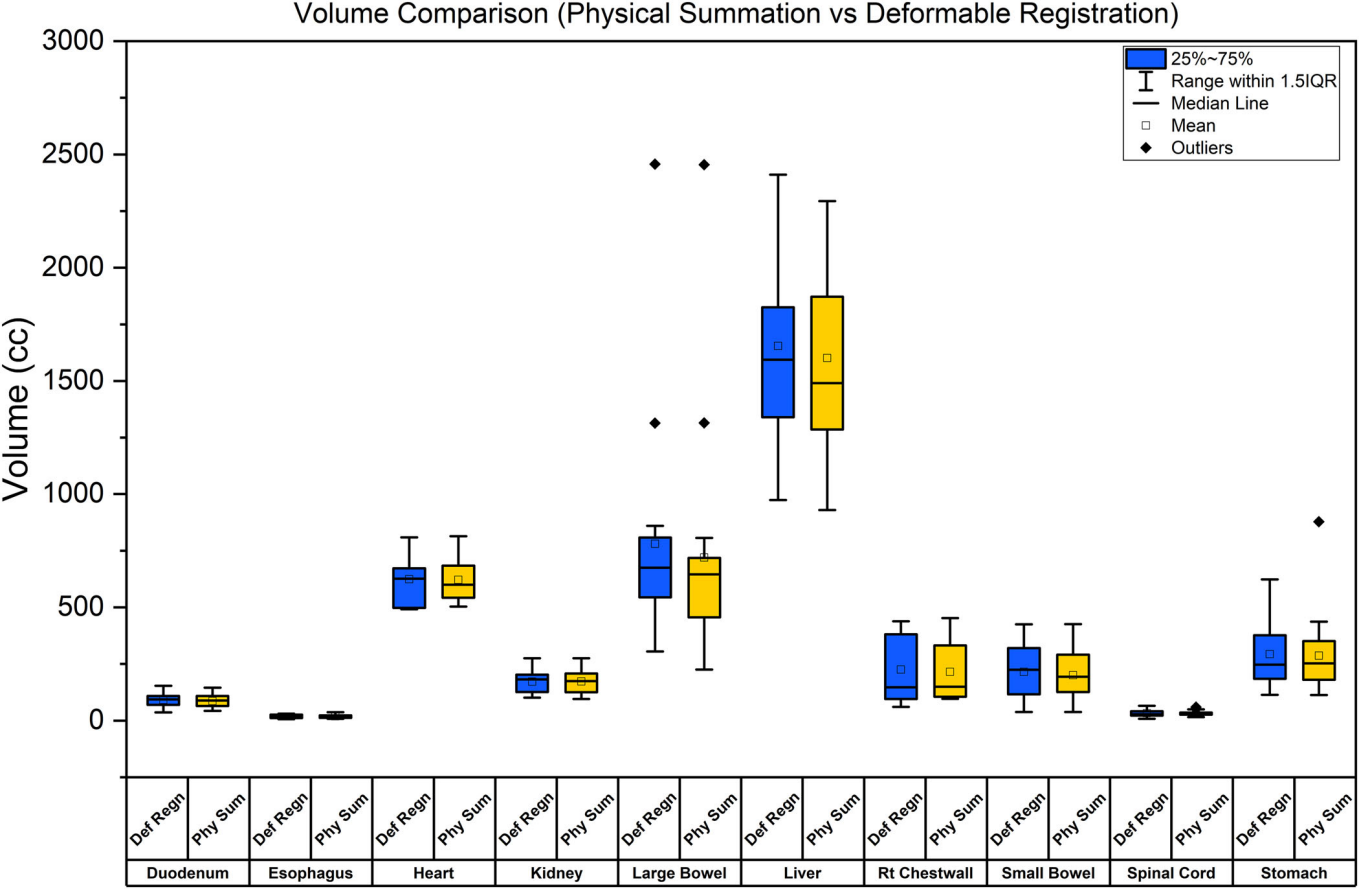
Comparison of organ volumes using Deformable Registration (Def Regn) and Physical Summation (Phy Sum) for various OARs. OARs, organs at risk.

Figure 2 illustrates the organ volumes estimated using DIR and physical summation methods. The findings demonstrated that organs susceptible to significant motion, such as the liver and large bowel, exhibited greater variability in volume measurements when evaluated using physical summation. In contrast, DIR, which accounts for anatomical deformations, provided more precise volume estimations. Rigid anatomical structures, such as the spinal cord and esophagus, showed minimal differences. However, the presence of whiskers and outliers in the box plots suggested that deformable registration is essential for accurately capturing anatomical changes in mobile organs, preventing both dose overestimation and underdosing.

### 3.3 Dose accumulation

**FIGURE 3:**
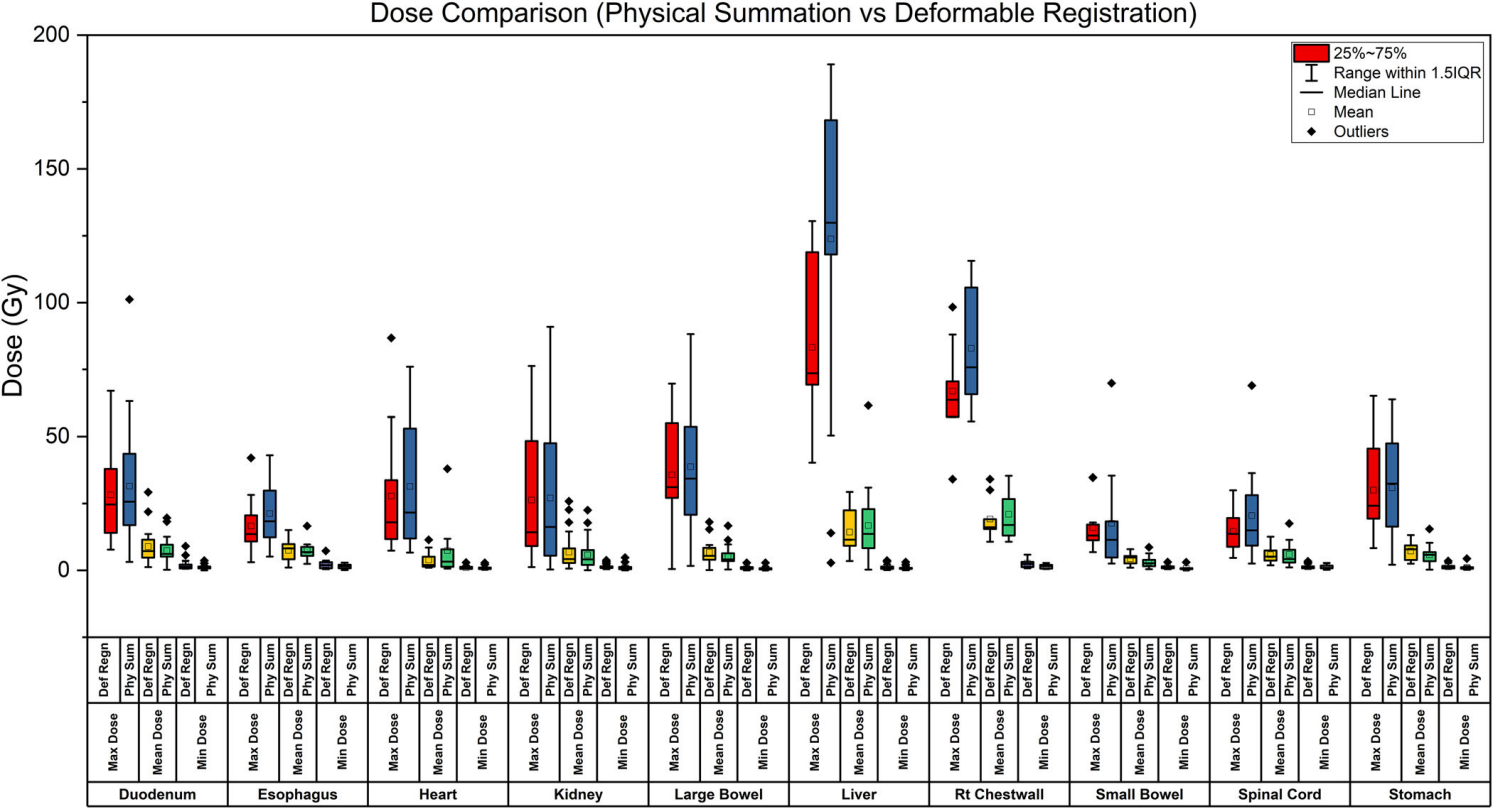
Comparison of maximum, mean, and minimum dose distributions across different organs using Deformable Registration (Def Regn) and Physical Summation (Phy Sum).

Figure 3 illustrates a comparative dose analysis between physical summation and DIR‐based dose accumulation, evaluating the maximum, mean, and minimum dose distributions across multiple anatomical structures. The results revealed that DIR‐based dose accumulation consistently provided lower maximum dose estimates compared to physical summation, particularly for highly mobile organs such as the liver and large bowel. This finding underscores the potential of DIR in preventing dose overestimation in these structures.

The mean dose values were comparable between both methods for most organs, including the esophagus and spinal cord, suggesting that both approaches yield similar results for relatively rigid anatomical structures. However, DIR yielded higher minimum dose estimates for organs such as the duodenum and stomach, likely due to its ability to account for positional variations, which are often underestimated in physical summation methods.

### 3.4 Comparative dosimetric analysis

Table 2 presents a statistical comparison of volume estimations and dose distributions between physical summation and DIR. A significant reduction in maximum dose for the liver was observed under deformable registration (83.2 ± 28.0 Gy) compared to physical summation (123.8 ± 55.6 Gy, *p* = 0.00), suggesting overestimation using physical summation. Similarly, differences were observed in the large bowel, stomach, and duodenum, where both minimum and mean doses were significantly higher under DIR (*p* < 0.05), reflecting better anatomical alignment across treatment courses. For the right chest wall, the maximum dose was significantly reduced under DIR‐based dose accumulation (66.9 ± 18.6 Gy vs. 82.9 ± 22.4 Gy, *p* = 0.05).

**TABLE 2:** Comparative analysis of physical summation versus deformable registration for OAR dose.

	Minimum Dose (Gy) (Average ± Std Dev)			Mean Dose (Gy) (Average ± Std Dev)			Maximum Dose (Gy) (Average ± Std Dev)		
Structures	Phy Sum	Def Regn	*p* value	Phy Sum	Def Regn	*p* value	Phy Sum	Def Regn	*p* value
Duodenum	1.2 ± 0.9	2.1 ± 2.2	**0.04**	7.4 ± 5.7	8.9 ± 7.5	**0.05**	31.5 ± 24.9	28.2 ± 17.5	0.26
Esophagus	1.5 ± 0.9	2.3 ± 1.9	0.17	7.5 ± 3.5	7.4 ± 4.0	0.82	21.2 ± 11.7	16.6 ± 10.6	0.09
Heart	1.0 ± 0.8	1.1 ± 0.8	0.80	7.4 ± 11.3	3.9 ± 3.6	0.36	31.4 ± 25.8	27.8 ± 25.6	0.22
Large bowel	0.7 ± 0.6	1.0 ± 0.6	**0.01**	5.4 ± 4.0	6.7 ± 4.5	**0.02**	38.7 ± 23.4	35.7 ± 19.4	0.31
Liver	0.9 ± 0.7	1.3 ± 1.0	**0.01**	16.8 ± 13.9	14.3 ± 7.4	0.41	123.8 ± 55.6	83.2 ± 28.0	**0.00**
Lt Kidney	1.0 ± 0.9	1.4 ± 1.0	**0.00**	4.0 ± 3.8	4.3 ± 3.2	0.60	15.5 ± 14.4	13.7 ± 11.7	0.39
Rt Chestwall	1.5 ± 0.8	2.7 ± 1.9	**0.04**	21.0 ± 9.7	19.2 ± 7.7	0.65	82.9 ± 22.4	66.9 ± 18.6	**0.05**
Rt Kidney	1.1 ± 1.2	1.4 ± 0.7	0.27	7.6 ± 6.3	9.5 ± 7.0	0.15	39.3 ± 29.3	39.7 ± 24.4	0.93
Small bowel	0.8 ± 0.7	1.2 ± 0.7	**0.00**	3.1 ± 2.4	4.4 ± 2.2	0.21	17.6 ± 19.0	16.0 ± 8.9	0.50
Spinal cord	1.2 ± 0.7	1.4 ± 0.8	0.37	5.9 ± 4.0	5.9 ± 3.0	0.89	20.5 ± 14.8	14.6 ± 7.3	0.08
Stomach	1.1 ± 0.9	1.3 ± 0.8	**0.04**	5.9 ± 3.4	7.2 ± 3.0	**0.03**	30.9 ± 16.9	30.0 ± 16.1	0.75

Abbreviation: OARs, organs at risk.

**FIGURE 4:**
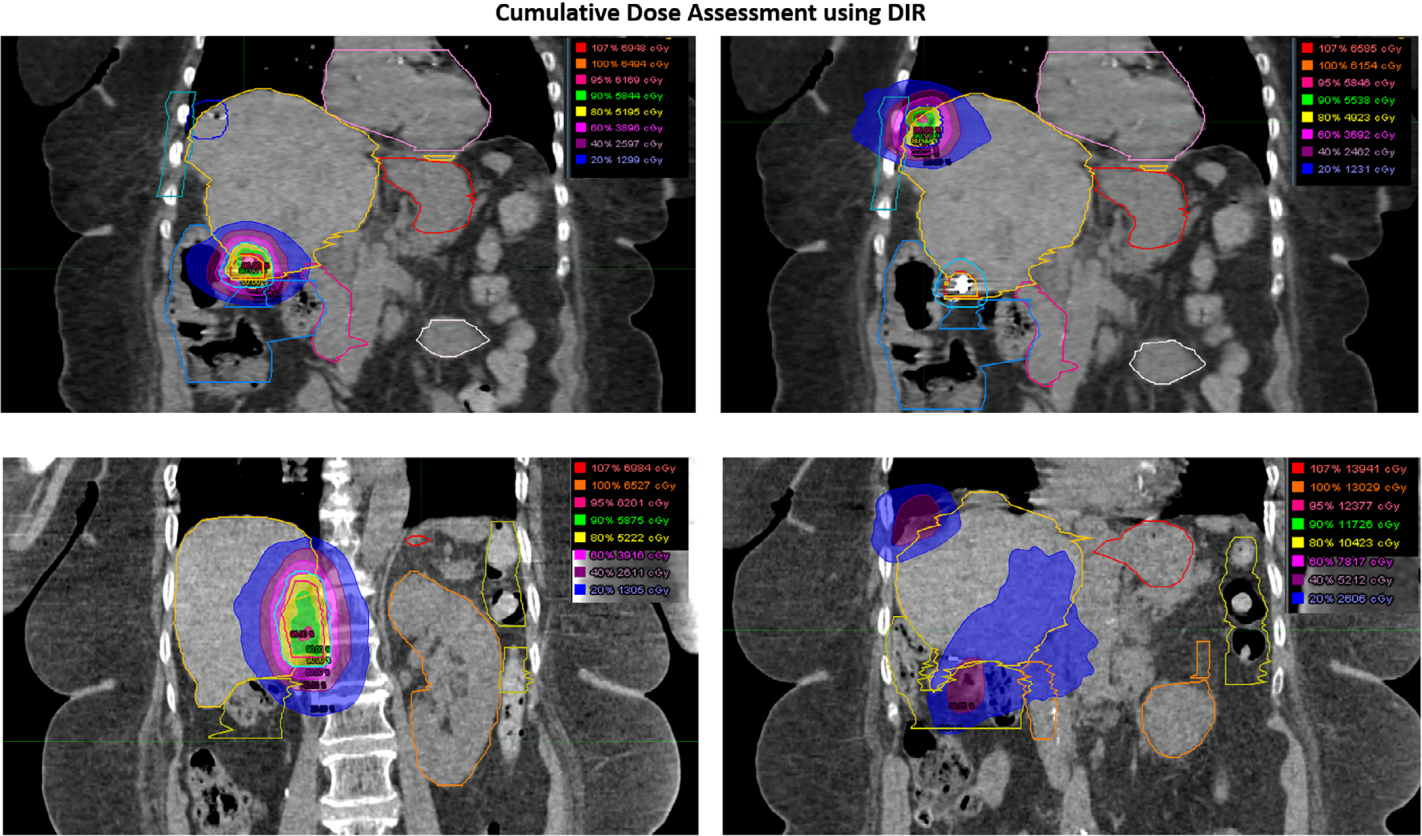
Illustration of dose distribution of a patient who received three stereotactic re‐irradiations using Robotic radiosurgery (Cyberknife SBRT) and the cumulative dose distribution generated using deformable registration.

With regard to mean dose, statistical significance was noted for the large bowel (*p* = 0.02), suggesting that DIR results in better dose optimization for this structure. Additionally, DIR significantly reduced the maximum dose in the liver (*p* = 0.00), highlighting its effectiveness in limiting high‐dose exposure to critical structures and reducing the risk of radiation‐induced toxicity. Figure 4 displays the qualitative illustration of a representative patient who underwent three courses of robotic stereotactic body radiotherapy (SBRT) and the cumulative dose distribution using the DIR for re‐irradiation of liver metastases.

### 3.5 DVH analysis

**FIGURE 5:**
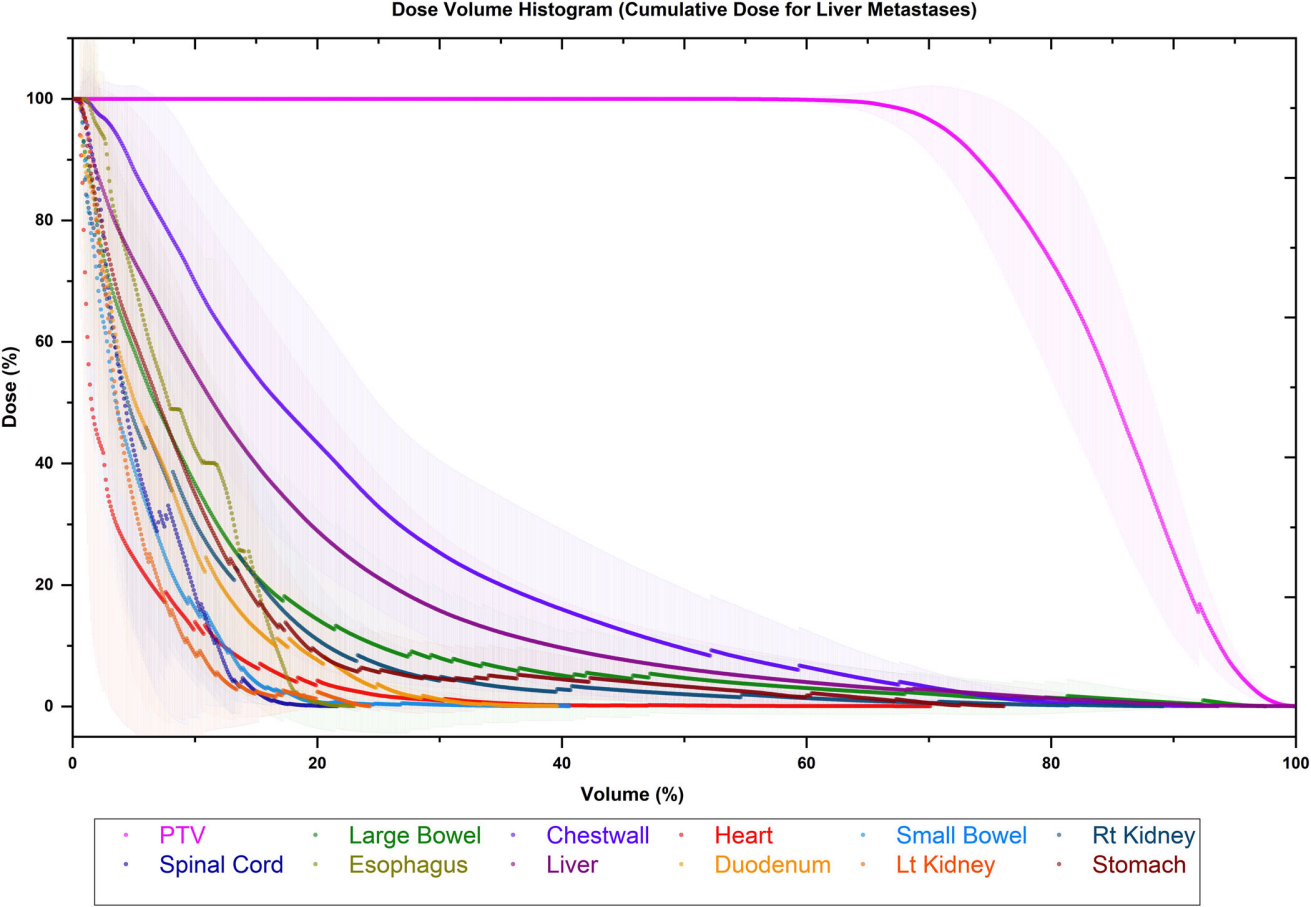
Cumulative DVH for various OARs using Deformable Registration Technique. DVH, dose‐volume histogram; OARs, organs at risk.

The DVH as shown in Figure 5 provides a quantitative assessment of cumulative dose distribution for the re‐irradiation of liver metastases, utilizing deformable registration techniques. This DVH illustrates mean accumulative doses with ranges to the target and a series of organs‐at‐risk (OARs), offering insight into dose coverage and sparing efficiency. The x‐axis indicates the percentage of volume, while the y‐axis denotes the dose received, presented as a percentage of the prescribed dose. This visualization aids in evaluating the therapeutic efficacy and safety of the applied re‐irradiation protocol.

The DVH for the OARs, including the liver, esophagus, spinal cord, and small and large bowels, reflects effective dose sparing. For instance, the spinal cord and heart curves are characterized by a rapid decline in dose percentages with increasing volume, indicative of their sparing from high radiation doses.

### 3.6 Biological dose summation using BED and EQD2

Table 3 presents the maximum dose, BED, and equivalent dose in 2 Gy fractions (EQD2) for various OARs in re‐irradiation settings. Each entry provides median values with ranges. The values across different organs provide insights into the varying radiation tolerances of these tissues and the biological impact of re‐irradiation on them.

**TABLE 3:** The maximum dose and biological dose measured using DIR based dose accumulation for various OARs after accounting for number of fractions.

Structure	Maximum dose	BED	EQD2
Cauda equina	36.31 (28.66–43.96)	52.71 (38.44–66.97)	31.62 (23.06–40.18)
Chestwall	66.25 (34.11–98.33)	185.91 (72.88–299.75)	111.55 (43.73–179.85)
Duodenum	27.45 (7.79–67.06)	53.55 (10.32–145.95)	32.13 (6.19–87.57)
Esophagus	13.25 (1.82–28.24)	18.75 (1.88–42.22)	11.20 (1.13–25.33)
Heart	27.79 (7.36–86.77)	74.90 (9.16 (9.16–400.47)	44.94 (5.50–240.28)
Large bowel	37.41 (13.22–69.70)	76.14 (19.05–194.25)	45.36 (11.43–116.55)
Liver	83.38 (40.25–130.47)	297.51 (75.80–753.20)	178.51 (45.48–451.92)
Lt Kidney	15.22 (2.15–54.90)	28.42 (2.40–166.52)	17.09 (1.44–99.91)
Ribs	54.38 (49.76–58.99)	133.02 (91.03–175.00)	79.81 (54.62–105.00)
Rt Kidney	38.15 (1.23–76.31)	92.80 (1.32–278.98)	55.63 (0.79–167.39)
Small bowel	14.30 (6.80–34.76)	21.10 (8.34–65.75)	12.66 (5.00–39.45)
Spinal cord	16.97 (4.67–62.70)	28.43 (5.03–193.73)	17.11 (3.02–116.24)
Stomach	29.84 (8.35–65.16)	54.59 (10.67–139.66)	34.55 (6.40–83.79)

Abbreviations: BED, biologically effective doses; DIR, deformable image registration; OAR, organs at risk.

For the cauda equina, the maximum dose is 36.31 Gy, with a corresponding BED of 52.71 Gy and EQD2 of 31.62 Gy. This suggests a moderate risk of damage due to the cauda equina's sensitivity to radiation. In contrast, the chest wall receives one of the highest doses, with a maximum of 66.25 Gy, a BED of 185.91 Gy, and an EQD2 of 111.55 Gy. These high values indicate the chest wall's potential for tolerating higher doses, but with caution due to the associated risk of complications such as rib fractures.

The maximum dose for the liver was observed as 83.38 Gy, a BED of 297.51 Gy, and an EQD2 of 178.51 Gy. Such doses reflect the liver's higher tolerance in specific re‐irradiation settings but also highlight the critical need for careful dose management to avoid radiation‐induced liver disease. The spinal cord received a maximum dose of 16.97 Gy, with a BED of 28.43 Gy and an EQD2 of 17.11 Gy, reflecting its critical sensitivity to even moderate doses of radiation. The variations in BED and EQD2 across organs help to tailor re‐irradiation protocols, ensuring that sensitive tissues are protected while delivering effective doses to target areas.

### 3.7 Quantitative assessment of deformable image registration for dose accumulation

**FIGURE 6:**
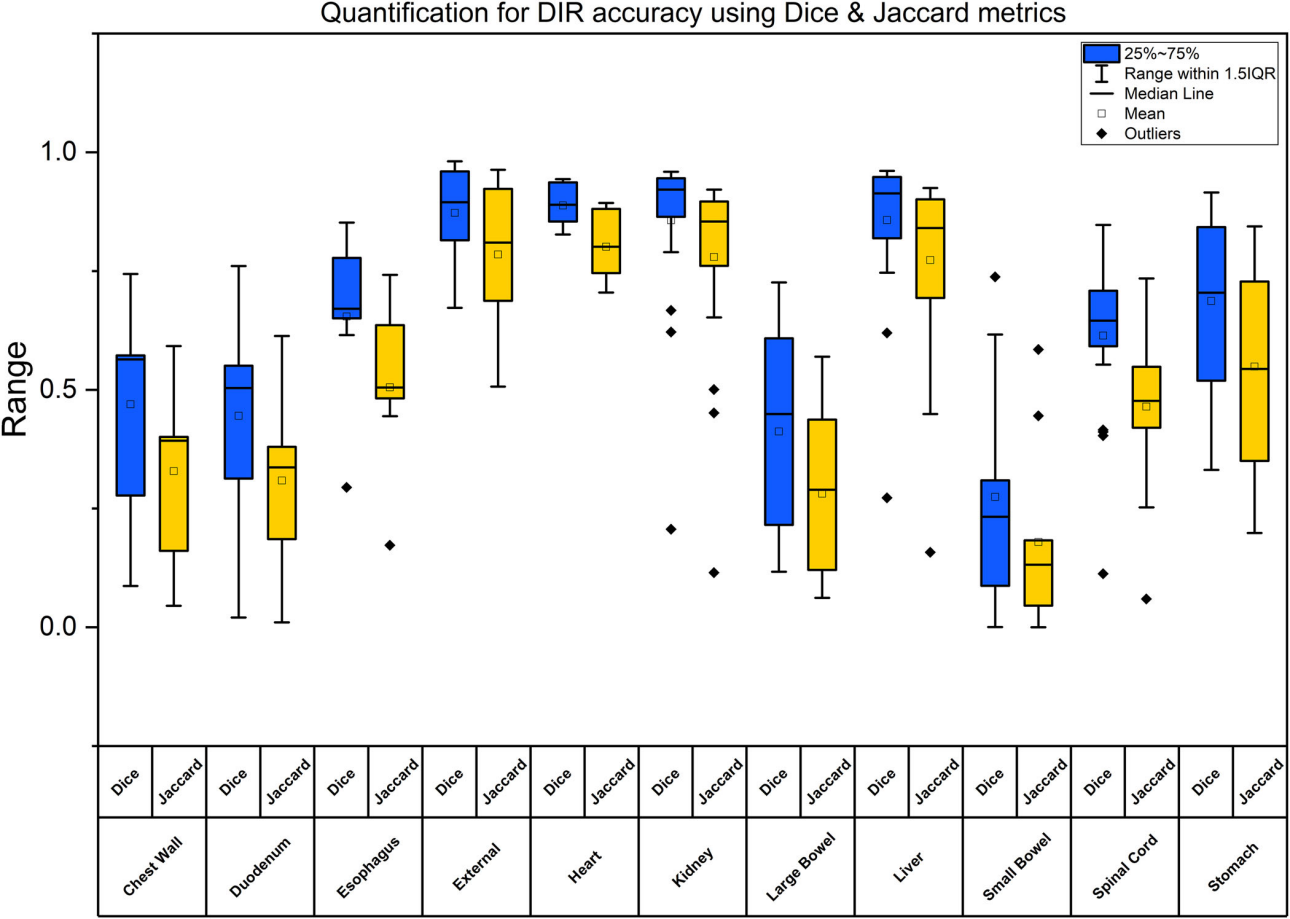
Quantitative evaluation of deformable registration using Dice coefficient and Jaccard index across various anatomical structures.

**FIGURE 7:**
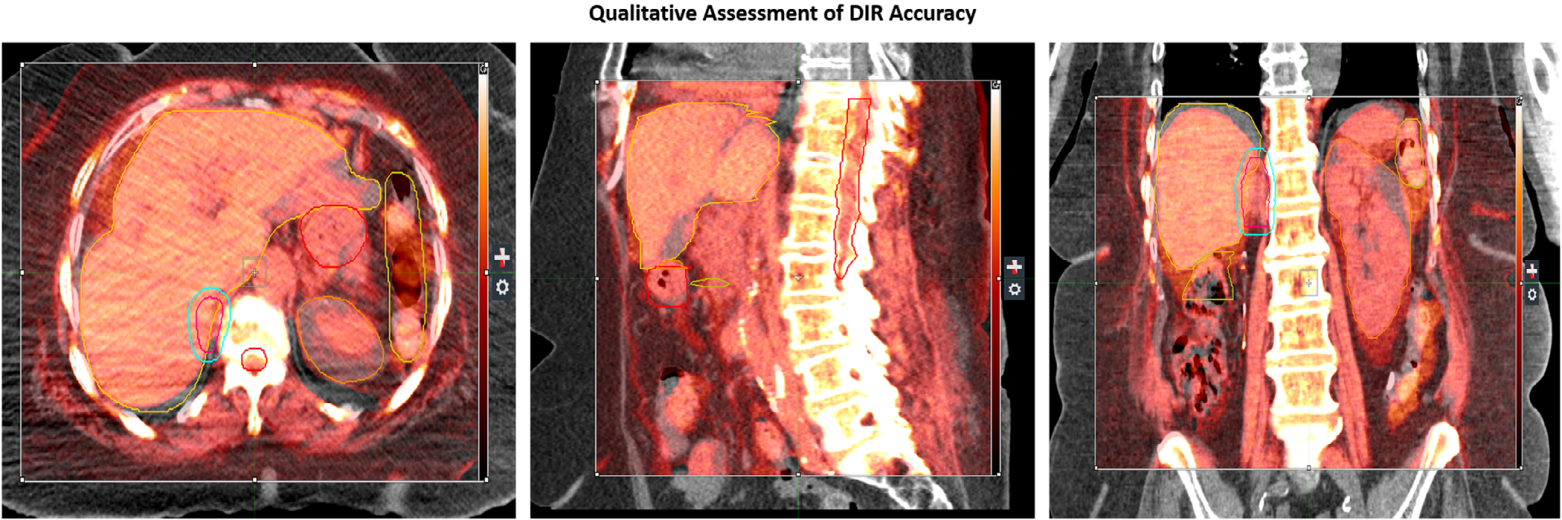
Illustration of DIR registration accuracy for a patient who underwent re‐irradiation of liver metastases using robotic radiosurgery. DIR, deformable image registration.

Figure 6 illustrates the quantitative evaluation of deformable registration across multiple anatomical structures using two distinct metrics for OARs. Among the analyzed structures, the heart demonstrated the highest spatial agreement, with a median Dice score of approximately 0.94 and a corresponding Jaccard index of 0.89. Similarly, the liver, being the primary target organ, exhibited excellent alignment with a median Dice score of 0.93 and a Jaccard index of 0.87. The external body also showed consistently high overlap metrics (Dice ≈ 0.91; Jaccard ≈ 0.84). In contrast, the small bowel presented the lowest similarity, with a median Dice score of 0.43 and Jaccard index of 0.29, likely due to its high deformability and positional variability. The spinal cord showed moderate agreement (Dice ≈ 0.70; Jaccard ≈ 0.56), while the duodenum and esophagus also exhibited moderate to low registration accuracy, with Dice scores ranging from 0.52 to 0.67 and Jaccard indices from 0.38 to 0.54. Structures such as the chest wall, heart, and esophagus exhibit higher Dice and Jaccard values, indicating good overlap and, therefore, higher registration accuracy. Conversely, organs like the spinal cord and small bowel show more variability, with larger IQRs and more outliers, indicating inconsistent registration in deforming regions. Figure 7 illustrates the DIR result demonstrating registration accuracy in a re‐irradiation case of liver metastases. The fused images (between two treatment) show that DIR achieved high alignment accuracy within the liver (DSC > 0.9), allowing precise mapping of previously irradiated regions onto the new planning dataset. However, registration accuracy was comparatively limited in the chest wall and large bowel (DSC ≈ 0.47), highlighting anatomical variability and challenges in deformable mapping outside the liver.

## 4 DISCUSSION

There is a lack of a widely recognized guideline for the dosage, fractionation, and volume when it comes to re‐irradiation. However, this practice has become increasingly common in recent times, often resulting in tolerable toxicity levels and improvements in local control or symptom management.^9^, ^39^, ^40^, ^41^, ^42^ The accuracy of dose accumulation in re‐irradiation is highly dependent on the selection of an appropriate image registration method, particularly for OARs affected by anatomical variations due to tumor progression, treatment‐induced changes, or physiological motion.^43^, ^44^

Deformable registration addresses these limitations by accommodating complex anatomical deformations, leading to enhanced dose distribution accuracy across treatment sessions.^45^ In this study, deformable registration demonstrated superior performance in aligning anatomical structures subjected to multiple re‐irradiation scenarios. DVHs indicated that deformable registration resulted in lower cumulative doses for moving organs, including the liver, bowel, and lungs, reflecting improved spatial alignment and dose conformity. For example, the liver's DVH revealed reduced cumulative doses with deformable registration, minimizing radiation‐induced toxicity.

A major challenge in evaluating the association between normal tissue and treatment‐related complications in re‐irradiation is the precise estimation of the actual dose absorbed by the tissue. To address this, biologically based dose metrics such as the BED and/or the equivalent dose in 2 Gy fractions (EQD2) are frequently utilized. BED, in particular, provides a theoretical basis for comparing various fractionation schedules.^46^ The Equivalent Dose in 2 Gy fractions, originally developed to estimate tumor control probability, represents the dose delivered in 2 Gy fractions that would produce an equivalent biological effect as a treatment delivered with a non‐standard fractionation scheme. Although its application to normal tissue effects has been questioned—primarily due to the underlying assumption of uniform dose distribution—EQD2 continues to be extensively employed in the assessment of treatment‐related toxicity. In practice, it is often used as a surrogate for BED in such analyses.^47^

The use of EQD2 for evaluating normal tissue dose in high‐dose‐per‐fraction SBRT regimens presents conceptual limitations. EQD2 assumes a standard 2 Gy per fraction scheme, which aligns with tumor dosing but does not reflect the variable dose‐per‐fraction often received by normal tissues. As a result, EQD2 becomes an abstract quantity when applied to normal tissues, lacking correspondence to actual treatment parameters such as true fraction number or dose per fraction. Although EQD2 is mathematically linked to the BED, BED itself is more directly representative of radiobiological response, being grounded in the cell survival model. Therefore, using BED or fraction‐specific biological dose models may offer a more practical and accurate alternative when assessing normal tissue effects in re‐irradiation and SBRT settings. Relying solely on EQD2 may lead to misinterpretation of biological risk, especially in scenarios involving multiple treatment courses and complex dose distributions.^48^

The study highlights the necessity of selecting registration methods based on the characteristics of the target and surrounding organs. These findings align with existing literature confirming that deformable registration significantly enhances dose accumulation accuracy during multiple re‐irradiation treatments of moving anatomical sites.^49^, ^50^ Importantly, all BED and EQD2 calculations were performed without accounting for the time intervals between the initial and subsequent treatments, which may lead to overestimation of the biological effects due to uncorrected tissue recovery. These findings highlight the necessity of personalized treatment planning, validated deformable image registration, and time‐corrected dose modeling to ensure safe and effective re‐irradiation.

The accuracy and reliability of deformable registration techniques were evaluated using established metrics, the Dice coefficient and Jaccard index. These metrics provide quantitative assessments of spatial alignment and overlap, crucial for verifying the performance of registration methods in clinical practice. Deformable registration consistently demonstrated superior accuracy, particularly for mobile and deformable tissues. The Dice coefficient and Jaccard index further validated the effectiveness of deformable registration, reflecting higher spatial overlap between registered images and their corresponding reference structures. This aligns with previous studies indicating that deformable registration achieves higher accuracy in aligning mobile organs, reducing geometric uncertainties.^51^, ^52^, ^53^ These results emphasize the importance of employing deformable registration for OARs with substantial deformation.

DVH analysis serves as a critical tool in comparing the effectiveness of registration methods in cumulative dose accumulation. The use of deformable registration techniques yielded a more realistic representation of dose distributions, as evidenced by the DVHs showing steeper declines in dose percentages with increasing volumes. This reflects better organ sparing and highlights the accuracy of deformable registration in accommodating anatomical variability. The superior performance of deformable registration in DVH analysis is consistent with other studies that reported that deformable registration reduced the cumulative dose exposure to the bowel and kidneys during re‐irradiation of abdominal tumors.^54^, ^55^, ^56^

### 4.1 Limitations

Despite the advantages of DIR, a few limitations were identified in this study. Variability in patient anatomy and treatment intervals posed challenges in maintaining consistent DIR accuracy, particularly in cases where significant anatomical changes occurred between treatment sessions due to tumor progression or regression. While DIR improved dose estimation for highly mobile organs, occasional misalignments occurred due to variation of scan lengths between the treatment plans, which needs to be verified manually before proceeding to deformable components.

Variability in imaging protocols and naming of structures within the plans may introduce variation in image registration, affecting the accuracy of dose estimation. Standardized imaging protocols and the use of standard nomenclature are essential for optimizing DIR performance in clinical practice. Another limitation is the lack of real‐time adaptation in current DIR methods. Since OARs undergo positional changes due to breathing and patient movement during treatment, integrating real‐time DIR adjustments could further enhance dose tracking and alignment accuracy. Further, MIM software is limited to five re‐irradiation treatment courses for deformable registration and dose accumulation.

The intensity‐based algorithm's limitations include the inability to register noisy CT images, image pairings with inconsistent Hounsfield Units, and image pairs with areas for which there is no relationship, including gas in the rectum and fecal matter. Image pairs should generally include the full exterior contour of the body for accurate findings at the image edge.^57^

Lastly, this study primarily focused on dosimetric accuracy without establishing direct correlations with long‐term clinical outcomes such as survival, tumor control, or toxicity reduction. Future research should incorporate clinical outcome assessments to validate the practical benefits of DIR‐based dose accumulation.

### 4.2 Future directions

Several areas warrant further investigation to refine DIR's application in dose accumulation for re‐irradiation. The integration of 4D imaging modalities, such as 4D CT and 4D MRI, may further improve DIR's ability to account for spatial and temporal anatomical changes, particularly for respiratory‐induced organ motion in the liver and lungs.

The application of artificial intelligence (AI) and machine learning could enhance DIR accuracy by automating registration processes and adapting to patient‐specific anatomical changes in real time. AI‐driven models could improve DIR performance by dynamically adjusting registration parameters based on deformation patterns, reducing the time and effort required for accurate dose accumulation.

Future research should also focus on long‐term clinical outcomes, correlating DIR accuracy with patient survival, tumor recurrence, and quality of life measures. Large‐scale, longitudinal studies are needed to confirm DIR's benefits beyond dosimetric accuracy and establish its role in improving patient outcomes. Additionally, personalized registration protocols should be explored, tailoring DIR parameters based on individual patient characteristics, such as organ elasticity and deformation patterns, to enhance accuracy and clinical effectiveness.

## 5 CONCLUSION

This study demonstrates that deformable image registration (DIR) significantly improves dose accumulation accuracy for robotic SABR‐based re‐irradiation in the management of liver metastases. DIR consistently supported in aligning OARs across multiple treatment sessions, leading to more accurate dose estimations, improved organ sparing, and reduced radiation‐induced toxicity. By providing a more realistic representation of dose accumulation, DIR mitigates the risks of overestimation and underestimation, enhancing the safety and efficacy of re‐irradiation treatments. Our findings demonstrate that integrating DIR into adaptive radiotherapy is crucial for improving treatment precision and minimizing OAR toxicity in multiple re‐irradiation settings.

## AUTHOR CONTRIBUTIONS

**Study conception and design**: All authors. **Data collection**: AB Mohamed Yoosuf, Mamdouh Alqathami, Salem AlShehri, MZA Aziz, and MS Mansor, GK Appalanaido. **Analysis and interpretation of results**: AB Mohamed Yoosuf, MZA Aziz, and MS Mansor. **Draft manuscript preparation**: AB Mohamed Yoosuf, Mamdouh Alqathami, Salem AlShehri, MZA Aziz, GK Appalanaido and MS Mansor. All authors reviewed the results and approved the final version of the manuscript.

## ETHICS STATEMENT

Ethical approval has been obtained from King Abdullah International Medical Research Center, Riyadh (Study No–NRC22R/648/12 & IRB Approval Number–IRB/0176/23).

## CONFLICT OF INTEREST STATEMENT

The authors declare no conflicts of interest.
